# Skeletal Muscle Cell Growth Alters the Lipid Composition of Extracellular Vesicles

**DOI:** 10.3390/membranes11080619

**Published:** 2021-08-12

**Authors:** Taylor R. Valentino, Blake D. Rule, C. Brooks Mobley, Mariana Nikolova-Karakashian, Ivan J. Vechetti

**Affiliations:** 1 Department of Physiology, College of Medicine, Lexington, KY 40536, USA; trvalentino@uky.edu (T.R.V.); c.brooks.mobley@uky.edu (C.B.M.); mariana.karakashian@uky.edu (M.N.-K.); 2 Department of Nutrition and Health Sciences, College of Education and Human Sciences, University of Nebraska-Lincoln, Lincoln, NE 68583, USA; brule2@unl.edu

**Keywords:** extracellular vesicles, lipids, muscle cell, muscle growth, EV biogenesis

## Abstract

We sought to characterize the lipid profile of skeletal muscle cell-derived Extracellular Vesicles (EVs) to determine if a hypertrophic stimulus would affect the lipid composition of C2C12 myotube-derived EVs. Analyses included C2C12 murine myoblasts differentiated into myotubes and treated with Insulin-Like Growth Factor 1 (IGF-1) for 24 h to induce hypertrophic growth. EVs were isolated from cell culture media, quantified using Nanoparticle Tracking Analysis (NTA) and analyzed using Transmission Electron Microscopy (TEM). EVs were homogenized and lipids extracted for quantification by Mass Spectrometry followed by downstream lipid class enrichment and lipid chain analysis. IGF-1 treatment elicited an increase in CD63 and CD81 levels (39% and 21%) compared to the controls (16%), respectively. Analysis revealed that skeletal muscle-derived EVs are enriched in bioactive lipids that are likely selectively incorporated into EVs during hypertrophic growth. IGF-1 treatment of myotubes had a significant impact on the levels of diacylglycerol (DG) and ceramide (Cer) in secreted EVs. Specifically, the proportion of unsaturated DG was two- to three-fold higher in EVs derived from IGF-treated cells, as compared to those from control cells. The levels of saturated DG were unaffected. Selective increases were similarly seen in C16- and C24-Cer but not in other species. Levels of free sphingoid bases tended to decrease, while those of sphingosine-1-phosphate was unaffected. Our results suggest that the lipid composition and biogenesis of skeletal muscle-derived EVs, are specific and highly selective during hypertrophic growth.

## 1. Introduction

It is well accepted that virtually all cells are capable of secreting minute vesicles into the extracellular space, a process that is conserved among bacteria, plants, and mammals [1,2,3]. These vesicles, collectively termed Extracellular Vesicles (EVs), can be categorized as either exosomes, microvesicles, or apoptotic bodies, depending on their size and biogenesis process [4]. The interest in EVs have grown exponentially following seminal work demonstrating that EVs can transfer functional cargo to recipient cells [5,6]. As the field evolves, the potential to utilize EVs as therapeutic delivery systems for drugs [7] and as biomarkers for physiological and pathological conditions [8] is becoming clear. In addition, understanding how EVs mediate effects on recipient cells provides a new means for intra/intercellular crosstalk.

It is generally perceived that EVs arise from specific pathways depending on their subtype [9,10]. For example, microvesicles are thought to shed off from the plasma membrane from cytoplasmic protrusions [11], while exosomes are formed from invaginations of endosomes, which give rise to intraluminal bodies/multivesicular bodies that fuse with the plasma membrane and are released into the extracellular space [12]. While the EV subtype nomenclature based on these mechanisms are well accepted, it has been argued that a subtype of exosomes could bud directly from the plasma membrane [13,14], suggesting that there may be non-canonical pathways of EV biogenesis. These alternative pathways might be dependent on a cellular type or perturbations. For example, in skeletal muscle cells, it has been demonstrated that RNA localization varies depending on the developmental stage of C2C12 myotubes and protein synthesis [15]. This exciting study indicates specific hubs for cellular processes and supports the notion that events in the cell do not take place randomly; hence, specific spatial locations serve a discrete purpose. These results might suggest that the cells engage in a particular pathway of EV biogenesis upon receiving a specific stimulus.

EVs are generally thought to contain biologically active cargo, such as mRNAs, miRNAs, proteins, and lipids, which mediate various effects on recipient cells [16]. A major focus in EV research has been identifying the miRNA content carried within EVs (exomiRs) [9,17,18]. Several studies have demonstrated that exomiRs are delivered to other cells, where they regulate their cognate target genes usually at the posttranscriptional level [19,20,21,22]. While these studies clearly demonstrated the functional transfer of miRNA, some studies have suggested that the modulation of gene expression in target cells via exomiRs may not account for all the biological properties of EVs [23]. For example, Chevillet and co-workers quantified the number of miRNAs from plasma, seminal fluid, dendritic cells, mast cells, and ovarian cancer cells and determined that there was less than one copy of a given miRNA per EV [24]. Helwa and co-workers further demonstrated the abundance of miRNA in EVs to be roughly one to two molecules of miRNA/EV [25]. This low abundance theory raises questions regarding the ability of exomiRs to alter the function of the recipient cells, since it was demonstrated that the typical concentration of a microRNA per cell, to effectively induce target silence, is estimated to be around 1000 copies [26]. Although this mechanism could be a cell-type and/or physiological/pathological status specific, it is interesting to speculate that there might be synergism among EV cargo to induce a biological effect into a recipient cell. Therefore, a better understanding of the various cargo within EVs could provide new information regarding their biological impact.

While exomiRs and proteins have been widely investigated, only recently has the lipid signature of EVs gained attention. Lipids are the most abundant component of the EV membrane bilayer, and it has been demonstrated that EVs are enriched for structural lipids, such as sphingomyelin, hexosylceramide, phosphatidylinositol, and free cholesterol [27,28,29]. In addition, lipids participate in EV biogenesis through various mechanisms, such as forming lipid rafts that concentrate or select cargo, altering membrane curvature, facilitating pitching from the multivesicular bodies, and participating in ESCRT-independent EV formation [30,31,32,33]. Furthermore, the lipids contained within EVs could be used as biomarkers for pathologies and/or to track their progression from their own respective biogenic pathway to the target cell(s) [34,35]. Specifically, EVs have been shown to contain bioactive lipids, such as ceramide (Cer) and diacylglyceride (DG) [27,36], which have been shown to be important for vesicular biogenesis, trafficking, and secretion [32,37,38,39,40]. These bioactive lipids may also play a role in the regulation of signaling pathways on the recipient cell, providing a possible mechanism of cell-to-cell crosstalk. Therefore, the lipid composition of EVs represents a new area of research not only for their role in EV biogenesis but also for their biological effect on the target cell(s) [31,41,42]. Most of the studies addressing exosome lipid content have been focused on structural lipids and show an enrichment from cells to exosomes of 2–3 times for cholesterol, sphingomyelin (SM), glycosphingolipids, and phosphatidylserine (PS), in a cell type-dependent manner. However, the levels of lipid metabolites with bioactive properties, such as DG, Cer, and sphingosine-1-phosphate (S1P), as far as one could ascertain, have not been investigated. A better understanding of whether EVs contain bioactive lipid metabolites, and if so what determine this content, is crucial for ultimately identifying the mechanism(s) by which these vesicles are formed and participate in intercellular communication.

Skeletal muscle accounts for roughly 40% of total body mass and is known to secrete a diverse spectrum of factors, collectively termed myokines, that participate in inter/intra cellular communication [43,44,45]. Moreover, skeletal muscle has been shown to release EVs carrying muscle-enriched miRNAs that can regulate skeletal muscle plasticity as well as recipient cells [46,47,48,49,50,51]. As noted previously, lipids may play a fundamental role in EV biology; however, data regarding lipids in skeletal muscle-derived EVs is lacking. We recently published data showing that skeletal muscle-derived EVs are secreted during muscle hypertrophy which subsequently target adipose tissue, resulting in an increase in lipolysis [22]. This is not seen under resting conditions, suggesting that during skeletal muscle hypertrophy, a subset of EVs is secreted, influencing the biological function of the recipient cell. One possible way to modulate the compositional features of EVs is to regulate the lipid composition incorporated into the membrane. Therefore, the purpose of this study was to characterize the lipid profile of skeletal muscle cell-derived EVs to determine if a hypertrophic stimulus would affect EV biogenesis. We hypothesized that a hypertrophic stimulus would affect the lipid composition of EVs compared to the control.

## 2. Materials and Methods

### 2.1. Cell Culture

The skeletal muscle cell line C2C12 (murine cell line from American Type Culture Collection, ATCC) myoblasts were grown in Dulbecco’s Modified Eagles Medium (DMEM) supplemented with 10% fetal bovine serum, 100 units/mL of penicillin, and 100 µg/mL of streptomycin at low confluence. To induce differentiation, growth medium was switched to DMEM supplemented with 2% horse serum, 100 units/mL of penicillin, and 100 µg/mL of streptomycin when cells were fully confluent. In order to remove contaminating myoblasts from the myotube cultures, differentiated myotubes were treated with 10 μm arabinosylcytosine (AraC; Sigma, St. Louis, MO, USA) for 24 h. Following treatment with AraC, cells were washed in phosphate-buffered saline (1X PBS) and transferred back into differentiation media for a 24 h recovery period prior to further treatments.

### 2.2. IGF-1 Treatment

Following 7 days of differentiation [52], C2C12 myotubes were treated with either PBS (Control) or recombinant IGF-1 (Treated, 200 ng/mL in serum/antibiotic-free media) for 24 h. After 24 h, the media were collected for EV isolation. This series of experiments were performed in 3 independent T150 cell culture flasks.

### 2.3. Extracellular Vesicle Isolation

EVs were isolated using Cushioned-Density Gradient Ultracentrifugation (C-DGUG) [53,54]. Briefly, ~40 mL of the serum/antibiotic-free media was centrifuged at 3000× *g* for 15 min to eliminate cell debris, followed by filtration (0.22 µm). The filtered media were added to a liquid cushion (60% Iodixanol) and ultracentrifuged at 150,000× *g* for 4 h at 4 °C to concentrate the small vesicles. Subsequently, these vesicles were resolved in a density gradient (40, 20, 10, and 5% iodixanol) and submitted to ultracentrifugation at 100,000× *g* for 18 h at 4 °C. The EV-rich, protein-low fractions (6 and 7) were then pooled and concentrated to 500 µL on an Amicon Ultra-4 Centrifugal Filter Unit with an Ultracel-10 membrane (MWCO = 10 kDa; Merck Millipore, Darmstadt, Germany, United States Affiliate) by centrifugation at 4000× *g* for 30 min.

### 2.4. Nanoparticle Tracking Analysis (NTA)

EV concentration and size distribution were determined using a NanoSight NS300 (Malvern Instruments Ltd., Worcestershire, UK) equipped with a 488 nm blue laser and a CMOS camera that allows for detection and quantification of small particles (~50–1000 nm). As the particles move via Brownian motion, their speed is relative to their size, which can be estimated by the Stokes–Einstein equation [55]. EV samples were diluted (1:1000) in Dulbecco’s phosphate-buffered saline (DPBS). Samples were captured at room temperature with automatic temperature monitoring. Each sample was captured 5 times at 60 s intervals (camera level 11). The resulting videos were analyzed with NTA software version 3.0 (detection threshold 5) where the mean values for concentration and size distribution were calculated.

### 2.5. Transmission Electron Microscopy (TEM)

A 30 µL droplet of purified EVs was placed on parafilm and the surface of a carbon-formvar-coated copper grid was placed upside down to make contact with the samples (~2 min). Afterward, the excess specimen was wicked away by touching a piece of filter paper to the edge of the grid surface, followed by 30 s of air drying. The grid was placed upside down onto a droplet of stained solution (1% phosphotungstic acid) for ~2 min. Excess fluid was wicked from the grid following staining, leaving a thin aqueous film on the surface, which was allowed to air dry. Samples were examined at 80 kV under a Hitachi H7500 TEM [56,57].

### 2.6. EV Detection with Exo-Check™ Antibody Array

A membrane-based antibody array (Exo-Check™, System Biosciences, Palo Alto, CA, USA) was used to detect 8 known EV markers (FLOT-1, ICAM1, ALIX, CD81, CD63, EpCAM, ANXA5, and TSG101), a GM130 cis-Golgi marker (cellular contamination control), a labeled positive control for HRP detection, and a blank spot as a background control, following the manufacturer’s instructions. First, the EV protein concentration was measured by a Qubit Protein assay kit (ThermoFisher, Waltham, MA, USA), using a Qubit Fluorometer 3.0 (ThermoFisher, Waltham, MA, USA). Next, 10 µg of pooled EV protein was lysed and incubated overnight in the antibody membrane array. Following the washing steps, the membrane was incubated with a detection buffer (Detection Reagent A + Detection Reagent B) and chemiluminescence was measured using a ChemiDoc MP System (Bio-Rad Laboratories). Band intensities were calculated with molecular imaging software (Image Lab, Bio-Rad Laboratories).

### 2.7. Lipid Extraction and Analyses

The lipid profile of whole myotube cells (*n* = 3/group) and their corresponding EVs (*n* = 3/group) were analyzed at the Lipidomics Core of the Medical University of South Carolina on a Thermo Fisher TSQ Quantum triple quadrupole mass spectrometer, operating in a multiple reaction monitoring positive ionization mode, as previously described [58]. Briefly, all EVs recovered from the same number of cells and the myotube cells were homogenized and aliquots of the homogenates equivalent to 1.5 mg of protein were injected with the appropriate internal standards (ISs). Lipids were extracted twice with a 2 mL ethyl acetate, isopropanol, and water (60:30:10 *v/v/v*) solvent. Lipid extracts were evaporated and reconstituted in 150 μL of 1 mM ammonium formate in 0.2% formic acid in methanol before being subjected to Liquid Chromatography–Mass Spectrometry (LC-MS/MS) on an HP1100/TSQ Quantum LC-MS/MS system. Samples were gradient-eluted from the BDS Hypersil C8, 150 mm × 3.2 mm, 3 μm particle size column, with a 1.0 mM methanolic ammonium formate per 2 mM aqueous ammonium formate mobile phase system. Peaks corresponding to the target analytes and ISs were collected and processed using the Xcalibur software system. Quantitative analysis was based on the calibration curves generated by spiking of an artificial matrix with the known amounts of the target analyte synthetic standards and an equal amount of the ISs. The target analyte/IS peak area ratios were plotted against the analyte concentration. The target analyte/IS peak area ratios from the samples were similarly normalized to their respective IS and compared with the calibration curves using a linear regression model. The results from this linear regression model were analyzed using lipidR software [59]. Briefly, lipid name parsing was performed to generate the lipid classes, chain lengths, and numbers of unsaturated bonds based on the LIPID MAPS Structure Database [60]. Following quality control, normalization was performed using probabilistic quotient normalization [61], followed by statistical analysis employing a linear model fit and calculating a moderated t statistic to identify the significantly different lipids between groups (control and IGF-treated) [62]. Differentially expressed genes were estimated using the empirical Bayes function with a false discovery rate (FDR) of 5%, using the Benjamini–Hochberg method.

### 2.8. Lipid Class Enrichment and Lipid Chain Analyses

A lipid set enrichment analysis was performed in the top lipids, based on fold-change, to detect preferential regulation of lipid classes, total chain lengths, or total unsaturation patterns between groups.

### 2.9. Statistical Analyses

All data are presented as the mean ± SE. Except for lipidomic data (statistical analyses discussed above), after determining normal distribution of the data using a Shapiro–Wilk test, a two-sided Student’s t-test was performed to test the effect of IGF-1 treatment on cells and EVs. Statistical analysis was performed with GraphPad Prism version 9.2.0 (GraphPad Software, San Diego, CA, USA). Statistical significance was set at *p* < 0.050.

## 3. Results

### 3.1. EV Characterization between IGF Treatment and Control

**Figure 1:**
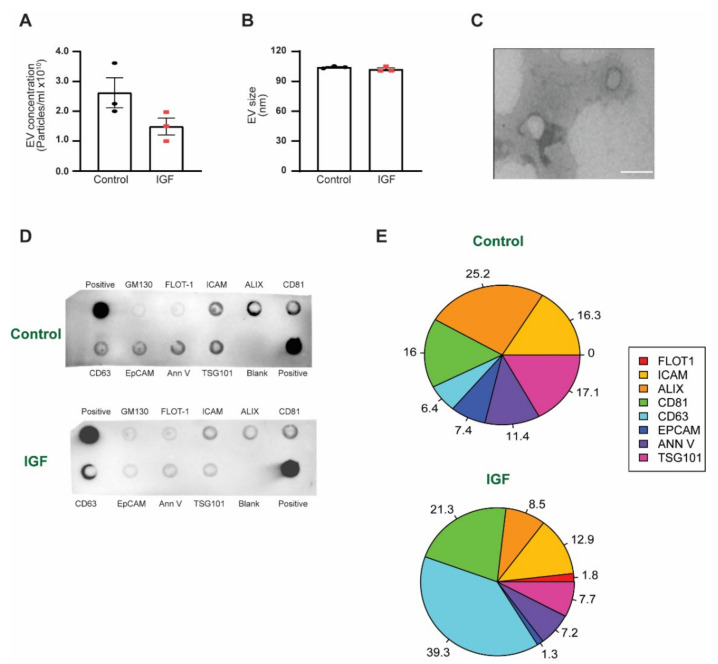
Characterization of myotube-derived EVs: (**A**)—myotube-derived EV control (control) and IGF-treated myotube-derived EV (IGF) concentration; (**B**)—EV size between the control and IGF; (**C**)—representative TEM image of the myotube-derived EVs, scale size 200 nm; (**D**)—EV marker protein concentration between the control and IGF; (**E**)—pie chart representing the percent of each EV protein markers in the control and IGF. (**A**,**B**) Data are presented as the mean ± SE (*n* = 3).

To determine if a hypertrophic stimulus (IGF-1 treatment) would impact EV biogenesis and release, we harvested EVs from the media of both the control and IGF-treated skeletal muscle cells and performed EV characterization. IGF treatment resulted in slightly lower levels of EV concentration compared to the control (Figure 1A). Additionally, there was no difference in size between the control and IGF-treated myotube-derived EVs (104.2 nm ± 1.10 nm and 102.13 nm ± 2.48 nm) (Figure 1B). The EV isolation procedures employed in this study resulted in a homogeneous population of EVs (characteristics similar to exosomes), as shown by TEM (Figure 1C), with specific EV surface markers and no cellular contamination (Figure 1D). Interestingly, the IGF-treated myotube-derived EVs were comprised of different levels of tetraspanin compared to the control (Figure 1E). Specifically, we found that EVs from the IGF-treated myotubes presented a substantial increase in CD63 levels compared to control EVs (39.3% vs. 6.4%, respectively). In addition, we observed that the most abundant protein markers found in the control EVs were ALIX (25%), followed by TSG101 (17%), ICAM, and CD81 (16%); whereas, in the IGF-treated myotube-derived EVs, CD63 was the most abundant, followed by CD81 (21%) and ALIX (8%).

### 3.2. Comparison of the Lipid Profiles between Myotubes and Myotube-Derived EVs

**Figure 2:**
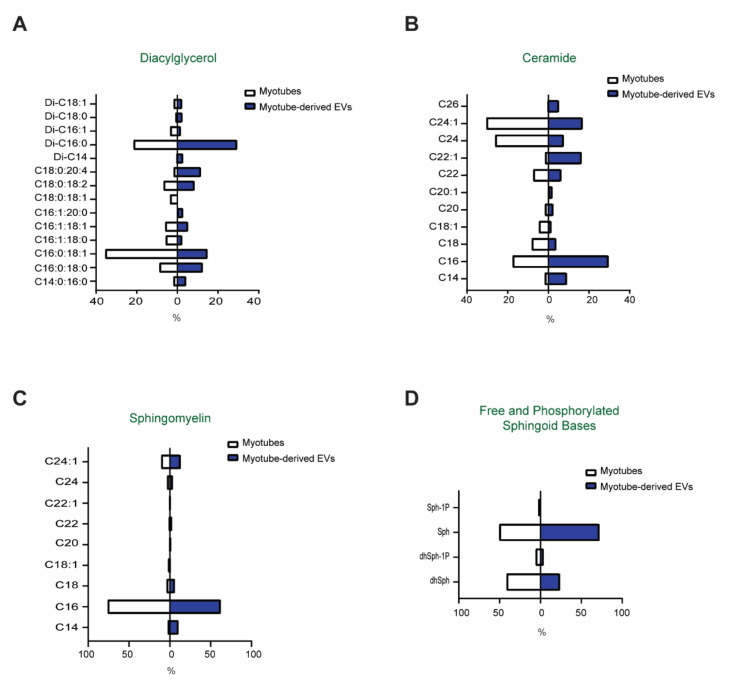
Lipid composition of the myotubes and their secreted EVs: (**A**)—diacylglycerol; (**B**)—sphingomyelin; (**C**)—ceramide; and (**D**)—free and phosphorylated sphingoid bases. Data are presented as percent enrichment in specific species (*n* = 3).

To test whether myotube-derived EVs contain bioactive lipids and how their bioactive lipid content compares to that of the donor cells, mass spectrometry-based analyses of the EVs and myotubes were performed. Specifically, with respect to SM, Cer, DG, and free (sphingosine (Sph) and dihydrosphingosine (dhSph)) and phosphorylated (S1P and dihydrosphingosine-1-phosphate (dhS1P)) sphingoid bases, as well as their respective phosphorylated bases (Figure 2A–D), were analyzed. As compared to myotubes, EVs were enriched in Di-C16-DG, as well as in DG containing arachidonic acid (C20:4), a product of Phospholipase C activation in the cells (Figure 2A). Ceramides containing C16 and C22:1 fatty acid were the major species in EVs, while in myotubes different Cer species, namely, C24:1 and C24:0, were most abundant (Figure 2B). As anticipated, SM is very abundant in EVs and the profile of SM species (i.e., length and saturation of the fatty acid attached to the C18:1 sphingoid base) was similar for the myotubes and EVs (Figure 2C). In both cases, C16 was by far the most abundant SM species, accounting for 60% of all sphingomyelins of the total SM content, followed by C24:1 (Figure 2C). The relative abundance of the free sphingoid bases (C18:0, sphinganine, and C18:1, sphingosine) as well as their phosphorylated forms, sphingosine-1-phosphate and sphinganine-1 phosphate, was similar for the myotubes and EVs (Figure 2D). Together, these results illustrate that (i) myotube-derived EVs carry a significant amount of bioactive lipids; and (ii) these bioactive lipids are most likely incorporated into the EVs through a process with some degree of selectivity, as they do not simply reflect the content of the cellular membranes of the donor cells.

### 3.3. Changes in Bioactive Lipid Profiles of EVs Following IGF Treatment of Myotubes

**Figure 3:**
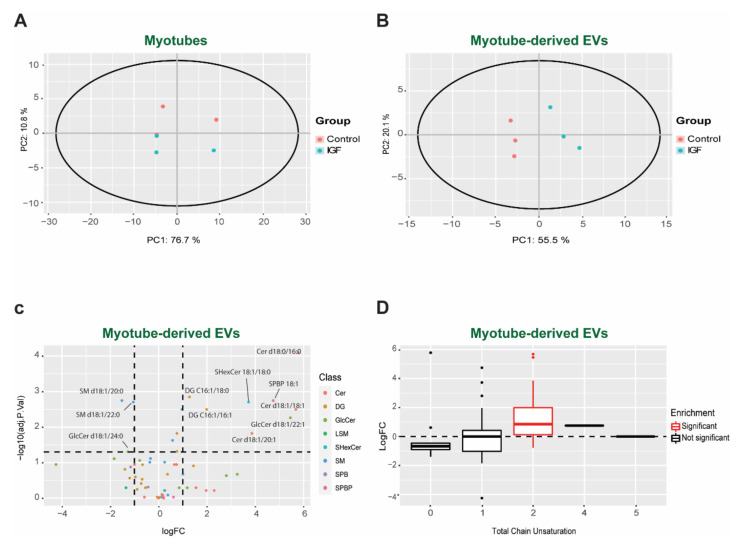
EV lipid profile characterization. Principal component analysis (PCA) plot between the control and IGF in myotubes (**A**) and myotube-derived EVs (**B**); (**C**)—volcano plot highlighting the significant lipid differences between the control and IGF EV; (**D**)—lipid chain plot revealing an increase in the degree of carbon chain saturation between the control and IGF EVs. (**C**,**D**) Data are represented as fold change in the IGF-treated myotube-derived EVs compared to the control. Significance is denoted (red) with an adjusted *p*-value < 0.05 (*n* = 3).

Having determined that EVs contain bioactive lipids, we then sought to test if this composition varies in different physiological conditions. The lipid profile from the control and IGF-treated myotubes and their corresponding EVs were analyzed by targeted LC-MS/MS. Unsupervised clustering analyses by principal component analysis (PCA) revealed similar patterns of the lipid composition between groups in myotubes. Interestingly, we observed a distinct cluster in myotube-derived EVs, represented by PC1 explaining 55.1% and PC2 explaining 20.1% of the variability, suggesting differences in lipid composition between groups (Figure 3A,B). Confirming our clustering analysis, we observed no statistical difference between the control and IGF-treated myotubes for DG (Figure S1), SM (Figure S2), and Cer (Figure S3). Despite no changes in the lipid profile of the myotubes, there was a distinct and significant different lipid profile in the EVs between each respective group (Figure 3C). Specifically, the IGF-1 treatment stimulated the release of EVs with higher levels of Cer (d18:0/16:0, d18:1/18:1, d18:1/20:1), DG (C16:1/18:0, C16:1/16:1), glucosylceramide (GlcCer, d18:1/22:1), Sulfatides Hexosyl ceramides (SHexCer, 18:1/18:0), and Sphingosine-1-phosphate (SPBP, 18:1), and lower levels of SM (d18:1/20:0, d18:1/22:0) and GlcCer (d18:1/24:0). Complementarily, our lipidomic analysis revealed a significant (adjusted *p*-value < 0.050) increase in unsaturation at position 2 of the carbon chains in the IGF-treated group compared to the control group (Figure 3D). Increasing the amount of unsaturated lipids in the membranes has been shown to increase their fluidity [63] and mitigates the unfolded protein response [64]. Therefore, adjusting the degree of unsaturation could be a way to increase the half-life and stabilization of the EVs before secretion.

### 3.4. IGF Treatment Exerts a Specific Impact on EV Lipid Composition

**Figure 4:**
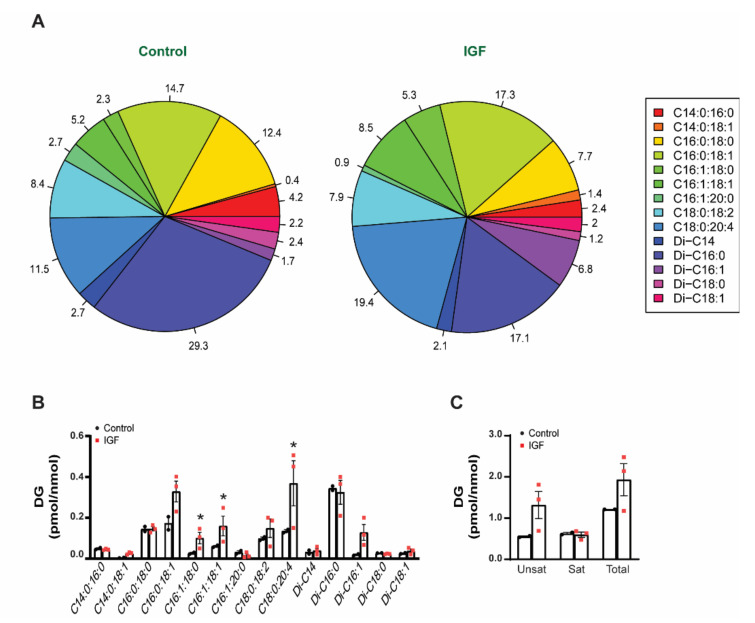
Effect of IGF-1 treatment on the diacylglycerol (DG) composition of myotube-derived EVs: (**A**)—pie chart highlighting the DG species in myotube-derived EV control and IGF-treated myotube-derived EVs; (**B**)—DG species normalized for the amount of lipid-associated phosphate (a measure of total phospholipids in each preparation); (**C**)—unsaturated, saturated, and total DG in control and IGF EVs. Data are presented in percentage (**A**) and as the mean ± SE (**B**,**C**) (*n* = 3), with significance denoted by an asterisk compared to the control.

Since we did not observe significant differences in the lipid profile of myotubes, the following analyses were focused on myotube-derived EVs. To assess the impact of IGF-1 treatment on the lipid profiles of EVs, the levels of individual lipid species were normalized for the amount of lipid-associated phosphate (a measure of total phospholipids in each preparation). Such normalization allowed us to discern changes that are specific for particular lipid classes from any changes that might be the result of an overall increase/decrease in total lipid content. These revealed that following IGF-1 treatment, the content of DG in the EVs increased by approximately 30% (Figure 4A). However, these increases were attributed to only a few specific species. Notably, dipalmitoyl-sn-glycerol (Di-C:16), which is the major DG species present in EVs, were not affected by IGF-1 treatment. Another major DG species containing saturated fatty acids only, stearoyl-palmitoyl-sn-glycerol (C:14-C:16), was similarly unaffected. However, the abundance of DG containing unsaturated fatty acids substantially increased following IGF treatment and the levels of C16:0-C18:1, C16:1-C18:0, C16:1-C18:1, C18:0–20:4, and Di-C16:1-DG doubled in the EVs derived from IGF-treated myotubes. Noteworthy, the C18:0-C20:4 levels tripled, demonstrating that IGF treatment had a greater effect on this particular DG species. (Figure 4B,C). Together, these data indicate that the IGF treatment of myotubes has a specific effect on the DG content of EVs, causing an increased abundance of DG with unsaturated fatty acids. The presence of selective changes also implies the involvement of lipid-metabolizing enzymes of distinct substrate specificity in the apparent remodeling of the DG content of EVs in response to hypertrophic conditions.

**Figure 5:**
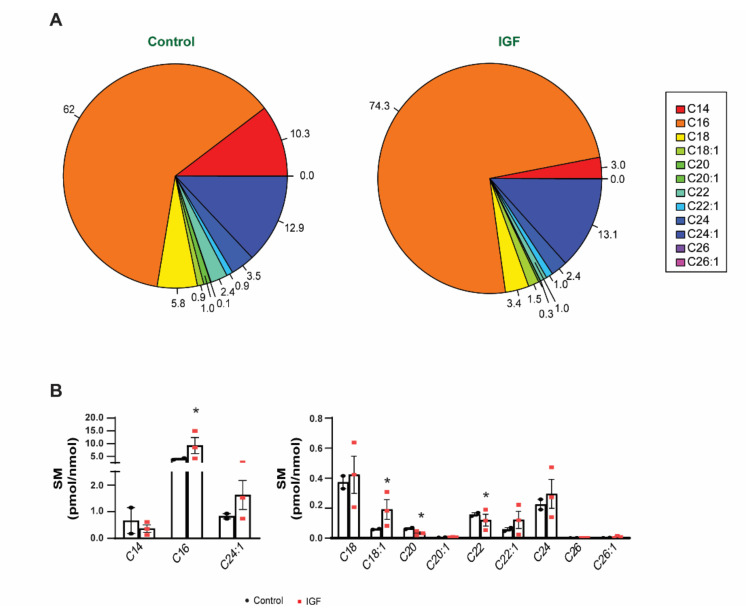
Effect of IGF-1 treatment on the sphingomyelin (SM) composition of myotube-derived EVs: (**A**)—pie chart highlighting the SM species in EVs derived from the control and IGF-treated myotubes; (**B**)—SM species normalized for the amount of lipid-associated phosphate (a measure of total phospholipids in each preparation). Data are presented in percentage (**A**) and as the mean ± SE (**B**) (*n* = 3), with significance denoted by an asterisk compared to the control.

We then analyzed the composition of SM, a structural lipid (Figure 5A). We observed a significant (adjusted *p*-value < 0.050) increase in C16 and C18:1 species in the IGF-treated myotube-derived EVs compared to control EVs. Furthermore, we found that IGF-1 treatment caused a significant reduction in C20 and C22 species compared to the control (Figure 5B). The length/saturation of the acyl chain in SM could contribute to the overall structure and/or function of EVs, by affecting the fluidity and stability of the lipid bilayer, as well as the affinity/strength of the interaction between SM and cholesterol. These results suggest another degree of selectivity in the generation of EVs during skeletal muscle growth.

**Figure 6:**
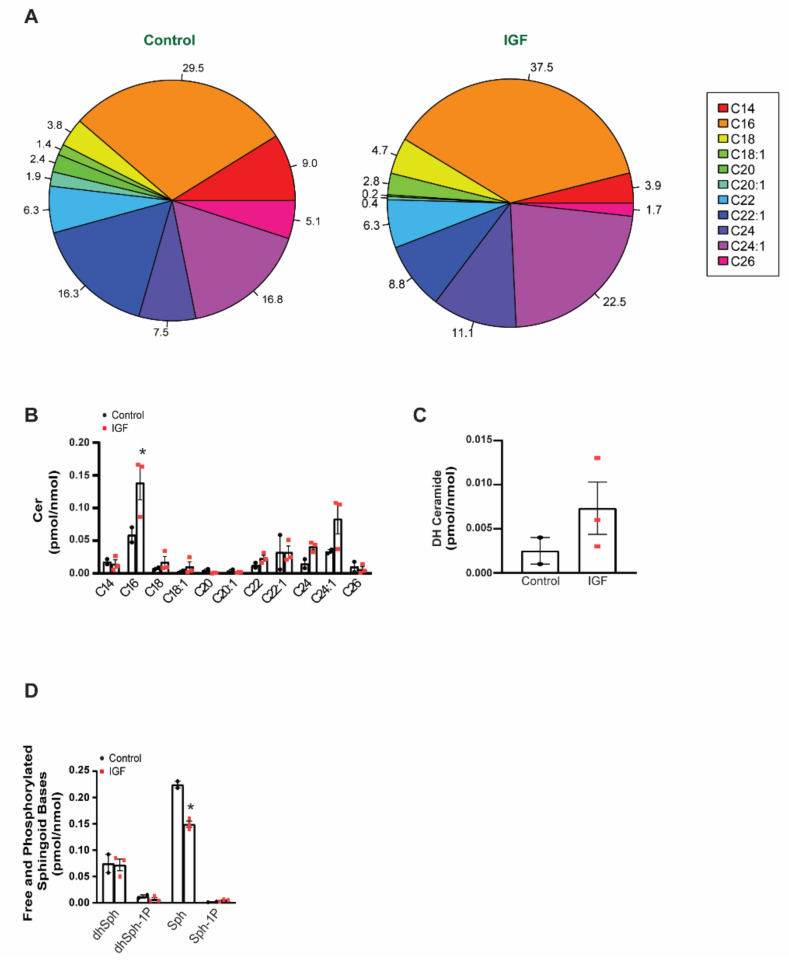
Effect of IGF-1 treatment on the ceramide (Cer) composition of myotube-derived EVs: (**A**)—pie chart highlighting the Cer species in EVs derived from the control and IGF-treated myotubes; (**B**)—Cer species normalized for the amount of lipid-associated phosphate (a measure of total phospholipids in each preparation). Total amount of dihydro-ceramide (DH) (**C**) and sphingoid bases (**D**) between the control and IGF EVs. dhSph: dihydrosphingosine; dhSph-1P: dihydrosphingosine-phosphate; Sph: sphingosine; Sph-1P: sphingosine-phosphate. Data are presented in percentage (**A**) and as the mean ± SE (**B**–**D**) (*n* = 3), with significance denoted by an asterisk compared to the control.

Similarly, we observed species-specific differences between the IGF-treated myotube-derived EVs and control EVs with regards to Cer (Figure 6A). The major species of Cer, namely, C16-, C24:1-, and C24:0-Cer, more than doubled following IGF treatment, while the minor species were unaffected. Interestingly, Cer can be produced by two metabolically distinct pathways, via hydrolysis of SM (mediated by sphingomyelinases, including neutral sphnogmyelianse-2, which is necessary for exosome secretion) or by the de novo pathway (mediated by Serine-Palmitoyltransferase and Cer synthases). Notwithstanding, the levels of dihydroceramide (DH-Cer) (Figure 6C), which is a precursor in the latter pathway, similarly increased, indicating possible involvement of de novo synthesis in the IGF-1-induced increase in EV Cer content. Furthermore, IGF-treated myotube-derived EVs had significantly (*p*-value < 0.050) less free sphingoid bases, particularly sphingosine, when compared to control EVs (Figure 6D). Taken together, these results suggest that skeletal muscle cell-derived EVs carry a significant amount of bioactive lipid metabolites and the fatty acid make-up of these bioactive lipids is strongly affected in conditions of muscle hypertrophy. Structural details in the DG and Cer molecules, including the length and saturation of the fatty acid, are not only introduced via distinct metabolic pathways, but also determine to a large extent the bioactive properties of these molecules. Therefore, our results provide strong evidence that the lipid content of the EVs released by a normal and hypertrophic muscle differ and may have a different impact on target cells.

## 4. Discussion

**Figure 7:**
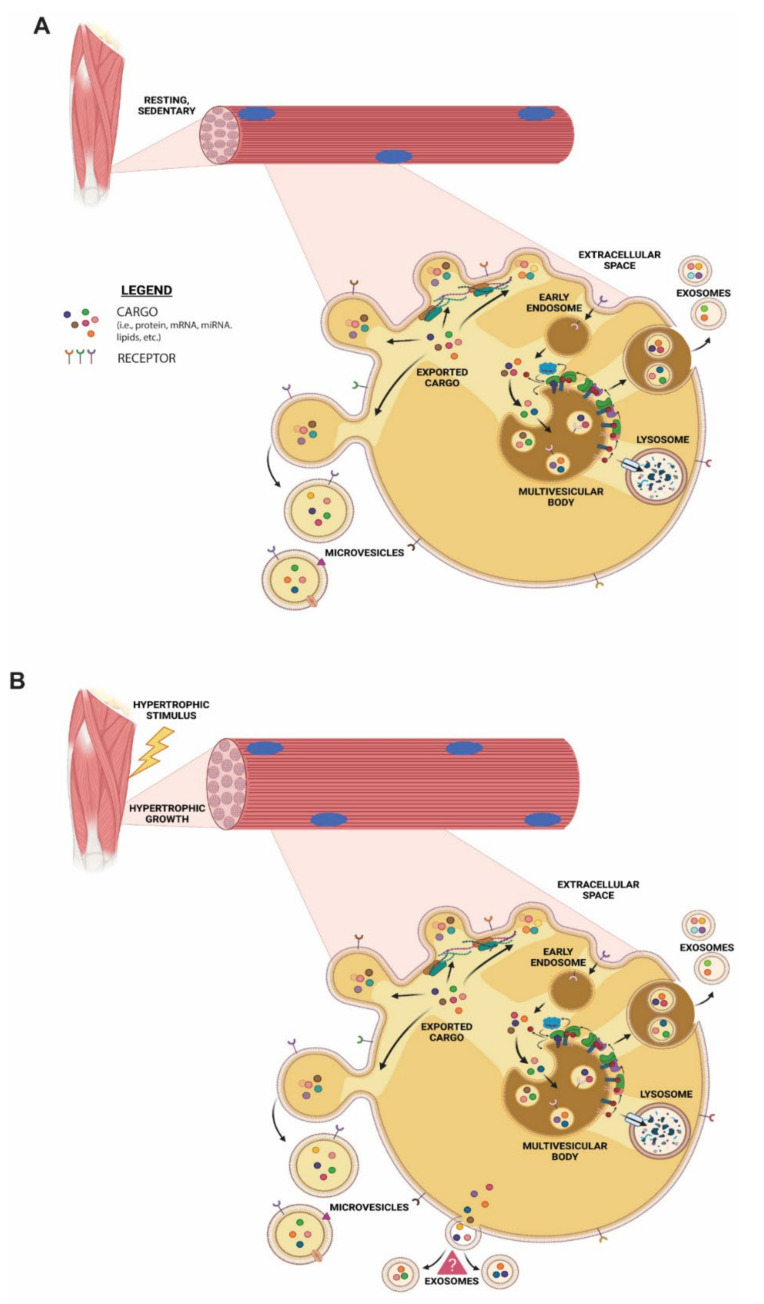
Proposed model of EV biogenesis in C2C12 myotube cells in resting conditions (**A**) and during muscle growth (**B**). In resting conditions, microvesicles and exosomes are formed through a canonical EV biogenesis mechanism, whereas during muscle growth an additional non-canonical exosome biogenesis mechanism might take place at the plasma membrane.

This study has provided evidence that a hypertrophic stimulus modulates EV biogenesis in skeletal muscle cells. Furthermore, by utilizing a robust method of EV isolation that yields a more homogeneous population (enrichment of exosomes), we demonstrated, for the first time, that myotube-derived EVs are enriched with specific bioactive lipids compared to that of the donor cells. Interestingly, our results demonstrate that the hypertrophic stimulus elicited by IGF-1 treatment alters the EV’s lipid composition, while having little effect on the composition of the donor cells. Specifically, IGF-treated myotube-derived EVs contained a substantial amount of unsaturated DG species compared to control EVs. Additionally, the EVs from the IGF-treated myotubes demonstrated specific increases in SM and Cer species, notably C16/C18 SM and C16 Cer. Interestingly, there was less sphingoid bases in the EVs from the IGF-treated myotubes, suggesting that Cer is preferentially incorporated into EVs over other sphingolipids. Skeletal muscle cell growth altered the EV marker proteins (i.e., CD63 and CD81), suggesting a different membrane composition is present in said EVs. Together, our results indicate that during skeletal muscle cell hypertrophy, exosome biogenesis could occur via a non-canonical pathway, possibly demonstrating that skeletal muscle cells have the capacity to utilize different pathways of EV biogenesis under the influence of specific stimuli (Figure 7).

Currently, the mechanisms associated with biogenesis aid in the classification of EV subtypes. For example, the most studied EV subtypes to date, microvesicles (ectosomes) and exosomes, are classified according to their biogenic processes. Specifically, microvesicles (ectosomes) are formed by budding off of the plasma membrane, while exosomes have been classically described as an ‘endosome-only’ mechanism [9,13,65]. However, recent research has suggested that exosome biogenesis may occur via a non-canonical pathway taking place at the plasma membrane [13]. In this elegant study, Gould’s group demonstrated that redirecting CD63 from endosomes to the plasma membrane increased its exosomal secretion 6-fold, whereas redirecting CD9 to endosomes reduced its exosomal secretion 5-fold [13]. The authors concluded that there is a common pathway that exosomes utilize for their biogenic process that occurs at both plasma and endosome membranes. A similar study conducted by Théry’s group made comparable observations; however, this group classified these vesicles as small ectosomes, since they were formed at the level of the plasma membrane [66]. Regardless of the nomenclature used, it is clear that EVs with similar characteristics of exosomes (size and classical markers) are formed at the plasma membrane. In addition, EV release fluctuates by utilizing different pathways, thus illustrating that the secretion of EVs is specific when comparing biogenesis and/or release from one compartment over the other.

Similar to these studies, we observed an enrichment in membrane-associated proteins in the IGF-treated myotube-derived EVs compared to control EVs. Specifically, our results demonstrate a substantial increase in CD63 and a slight increase in CD81 levels following IGF-1 treatment. Additionally, we detected a significant decrease in the amount of EVs secreted using a hypertrophic stimulus. Although limited, these results suggest that during skeletal muscle growth, a distinct EV population is preferentially formed and released. Even though isolation of a single population of EVs is challenging, in our study, the isolation method resulted in a homogeneous population of EVs (size and morphology) with similar characteristics of exosomes rather than a consortium of vesicles. Therefore, we propose that myotubes, under a hypertrophic stimulus, preferentially produce EVs (most likely exosomes) at the plasma membrane. While additional experiments are needed to confirm/refute these findings, this possibility presents exciting new areas for skeletal muscle EV research.

Aside from tetraspanin differences, which may suggest spatial differences in EV biogenesis, our lipidomic analysis highlights EV heterogeneity between the IGF-treated myotube-derived EVs and control EVs. Lipids are among the most abundant molecules within EVs with essential roles in structure (membrane stability and rigidity), formation, and release, and are potent modulators of cell signaling. Interestingly, the lipid incorporation in EV membranes seems to be a highly selective mechanism, as the lipid composition does not mirror the membrane composition of the donor cell(s). In fact, differences in lipid composition between EVs and the respective donor cells have been previously demonstrated [27,67]. Comparable to these studies, our results also demonstrate a specificity in EV lipid composition with an enrichment of SM and Cer in EVs when compared to myotubes. While both lipid species have been shown to participate in EV biogenesis and membrane organization [68,69], their roles in EV biology are not fully characterized.

Despite the differences between EVs and muscle cells, we did not observe any difference in the lipid composition in myotubes between groups. Curiously, our results suggest that a hypertrophic stimulus induces a distinct lipid profile in the EVs derived from growing myotubes. Differences in the lipid profiles of EVs have already been identified between cancer and non-cancerous cells [70], indicating that EV biogenesis is not static but rather a dynamic mechanism that is cell type/perturbation dependent. One of the most striking changes observed in our study was the significant enrichment of DG. Specifically, the levels of unsaturated C16:0-C18:1, C16:1-C18:0, C18:1-C16:1, and Di-C16:1 doubled, while C16:0-C20:4 nearly tripled in the IGF-treated myotube-derived EVs compared to control EVs (Figure 3A–C). DG is present in the plasma membrane and is considered a bioactive lipid, representing a major secondary messenger in addition to contributing to membrane curvature and fusion [71]. Consequently, the incorporation of DG into the EVs from IGF-treated myotubes may provide these vesicles with certain characteristics that enable them to target specific recipient cells. The increases seen in palmitoyl-arachidonoyl (C16:0-C20:4)-sn-gycerol is particularly interesting because, on one hand, this type of DG is produced selectively via activation of Phospholipase C, while, on the other hand, it contains the lipid precursor for the synthesis of prostaglandins and leukotrienes (arachidonic acid). Additionally, the packaging of DG in EVs under a hypertrophic stimulus could be a means by which skeletal muscle enables itself to become more insulin sensitive. DGs can induce insulin resistance in skeletal muscle by activating PCK-ϴ, impairing the insulin receptor substrates’ ability to activate PI3 kinase [72]. In support of this idea, it has been shown that DG species are highly correlated with insulin resistance and significantly lower in athletes [73]. Therefore, the release of DG via EV secretion after a hypertrophic stimulus could allow muscle cells to become transiently more inulin sensitive, resulting in higher glucose uptake. Alternatively, myotube-derived EVs carrying high amounts of DG to the target tissues may transiently decrease the insulin sensitivity in the recipient cells, allowing skeletal muscle to consume the majority of the circulating glucose. Although possible, the exact mechanism of DG in EV biology requires further investigation. Regardless, our results demonstrated that under a hypertrophic stimulus, specific DG species become highly enriched in EVs, therefore suggesting a specific function during skeletal muscle growth.

In addition to increases in specific DG species, we report that C16-, C24:1- Cer, and dihydroceramide are significantly elevated in EVs from IGF-treated myotubes. Ceramides are precursors to sphingolipids, produced from a four-step cascade that begins with the condensation of serine and palmitoyl-CoA [74]. Previously, it was found that Cer plays a role in EV biogenesis by interacting with endosomes and lysosomes, initiating an ESCRT-independent pathway [32,75] via nSMase2, which is essential for exosome biogenesis in some cells [76]. We previously found that mice treated with an nSmase2 inhibitor (GW4869) had a significant decrease in EV concentration [22]. Cer is also important for mediating the EV response in the recipient cell, which suggests that this particular lipid adds to the functionality of EVs. For example, EV-specific Cer can be incorporated into lipid rafts, which has the potential to regulate the signaling cascades in a recipient cell [23]. It is interesting to note that Cer in skeletal muscle, specifically C16, is associated with insulin resistance [77] and impairs myogenic differentiation in C2C12 myotubes [78]. C16 Cer has also been shown to specifically interact with P53 with its central DNA-binding domain, promoting P53 stabilization [79]. These authors reported that the binding of C16 Cer to P53 increased during starvation, demonstrating that C16 increases the pro-apoptotic pathways during periods of stress [79]. Therefore, the release of C16 Cer in EVs from muscle cells during hypertrophy could be the means by which muscle communicates with other tissues to downregulate or alter their metabolism. Conceptually, this would ensure that muscle consumes the appropriate amount of energy required for anabolism. Furthermore, this idea is in agreement with our previous work, where we show that EVs secreted by skeletal muscle during mechanical overload target adipose tissue, resulting in an increase in lipolysis [22]. This could be a mechanism whereby growing skeletal muscle signals to adipose tissue to increase the triacylglycerides that would support hypertrophic growth. Future studies are warranted and will need to determine the importance of this lipid in skeletal muscle cell-derived exosomes and their effect on systemic tissues.

The present study is not without its limitations. While our data provide specific information on skeletal muscle cell-derived EVs, our in vitro model of hypertrophy only indirectly reflects the mechanism(s) that are seen using various in vivo skeletal muscle hypertrophy models. Additionally, while our isolation method should yield a more homogeneous population of EVs (mainly exosomes), we cannot discard the possibility that small vesicles with the same density were co-isolated with our current population of EVs. Thus, we have chosen to characterize our vesicles as EVs rather than a specific subtype. Finally, we do not provide any downstream target cell-to-cell interaction analysis to determine the impact of skeletal muscle cell-derived EVs on the recipient cell and cannot conclude that EVs from the control or IGF-treated myotubes specifically affect systemic tissues.

## 5. Conclusions

In conclusion, this study provides the first evidence that demonstrates that a hypertrophic stimulus affects the composition of lipids in EVs. In addition, we show that the lipid composition differs between the parent cell and EVs, suggesting the lipid selectivity of the EVs is highly regulated. We also present initial findings that suggest that under a hypertrophic stimulus the biogenesis of EVs may preferentially occur at a different cellular location. The implications of these data could greatly improve the ability to identify pathways for EV biogenesis and subsequently track EVs from skeletal muscle after exercise. Finally, the lipid composition of myotube-derived EVs may provide information that pertains to the function of these EVs in the recipient cell.

## Supplementary Materials

The following are available online at https://www.mdpi.com/article/10.3390/membranes11080619/s1, Figure S1: Diacylglycerol (DG) composition of C2C12 myotube cells; Figure S2: Sphingomyelin (SM) composition of C2C12 myotube cells; Figure S3: Ceramide (Cer) composition of C2C12 myotube cells.

## References

[1] Choi Y., Kwon Y., Kim D.K., Jeon J., Jang S.C., Wang T., Ban M., Kim M.H., Jeon S.G., Kim M.S. (2015). Gut microbe-derived extracellular vesicles induce insulin resistance, thereby impairing glucose metabolism in skeletal muscle. Sci. Rep..

[2] Pitt J.M., Kroemer G., Zitvogel L. (2016). Extracellular vesicles: Masters of intercellular communication and potential clinical interventions. J. Clin. Investig..

[3] Rome S. (2019). Biological properties of plant-derived extracellular vesicles. Food Funct..

[4] Thery C., Witwer K.W., Aikawa E., Alcaraz M.J., Anderson J.D., Andriantsitohaina R., Antoniou A., Arab T., Archer F., Atkin-Smith G.K. (2018). Minimal information for studies of extracellular vesicles 2018 (MISEV2018): A position statement of the International Society for Extracellular Vesicles and update of the MISEV2014 guidelines. J. Extracell. Vesicles.

[5] Valadi H., Ekstrom K., Bossios A., Sjostrand M., Lee J.J., Lotvall J.O. (2007). Exosome-mediated transfer of mRNAs and microRNAs is a novel mechanism of genetic exchange between cells. Nat. Cell Biol..

[6] Ratajczak J., Wysoczynski M., Hayek F., Janowska-Wieczorek A., Ratajczak M.Z. (2006). Membrane-derived microvesicles: Important and underappreciated mediators of cell-to-cell communication. Leukemia.

[7] Vader P., Mol E.A., Pasterkamp G., Schiffelers R.M. (2016). Extracellular vesicles for drug delivery. Adv. Drug Deliv. Rev..

[8] Szabo G., Momen-Heravi F. (2017). Extracellular vesicles in liver disease and potential as biomarkers and therapeutic targets. Nat. Rev. Gastroenterol. Hepatol..

[9] Vechetti I.J., Valentino T., Mobley C.B., McCarthy J.J. (2020). The role of extracellular vesicles in skeletal muscle and systematic adaptation to exercise. J. Physiol..

[10] Heijnen H.F., Schiel A.E., Fijnheer R., Geuze H.J., Sixma J.J. (1999). Activated platelets release two types of membrane vesicles: Microvesicles by surface shedding and exosomes derived from exocytosis of multivesicular bodies and alpha-granules. Blood.

[11] Cocucci E., Racchetti G., Podini P., Meldolesi J. (2007). Enlargeosome traffic: Exocytosis triggered by various signals is followed by endocytosis, membrane shedding or both. Traffic.

[12] Camussi G., Deregibus M.C., Bruno S., Cantaluppi V., Biancone L. (2010). Exosomes/microvesicles as a mechanism of cell-to-cell communication. Kidney Int..

[13] Fordjour F.K., Daaboul G.G., Gould S.J. (2019). A shared pathway of exosome biogenesis operates at plasma and endosome membranes. bioRxiv.

[14] Booth A.M., Fang Y., Fallon J.K., Yang J.M., Hildreth J.E., Gould S.J. (2006). Exosomes and HIV Gag bud from endosome-like domains of the T cell plasma membrane. J. Cell Biol..

[15] Denes L.T., Kelley C.P., Wang E.T. (2021). Microtubule-based Transport is Essential to Distribute RNA and Nascent Protein in Skeletal Muscle. bioRxiv.

[16] Yoon Y.J., Kim O.Y., Gho Y.S. (2014). Extracellular vesicles as emerging intercellular communicasomes. BMB Rep..

[17] Bhome R., Del Vecchio F., Lee G.H., Bullock M.D., Primrose J.N., Sayan A.E., Mirnezami A.H. (2018). Exosomal microRNAs (exomiRs): Small molecules with a big role in cancer. Cancer Lett..

[18] Cheng L., Sharples R.A., Scicluna B.J., Hill A.F. (2014). Exosomes provide a protective and enriched source of miRNA for biomarker profiling compared to intracellular and cell-free blood. J. Extracell. Vesicles.

[19] Haneklaus M., Gerlic M., Kurowska-Stolarska M., Rainey A.A., Pich D., McInnes I.B., Hammerschmidt W., O’Neill L.A., Masters S.L. (2012). Cutting edge: miR-223 and EBV miR-BART15 regulate the NLRP3 inflammasome and IL-1beta production. J. Immunol..

[20] Montecalvo A., Larregina A.T., Shufesky W.J., Stolz D.B., Sullivan M.L., Karlsson J.M., Baty C.J., Gibson G.A., Erdos G., Wang Z. (2012). Mechanism of transfer of functional microRNAs between mouse dendritic cells via exosomes. Blood.

[21] Pegtel D.M., Cosmopoulos K., Thorley-Lawson D.A., van Eijndhoven M.A., Hopmans E.S., Lindenberg J.L., de Gruijl T.D., Wurdinger T., Middeldorp J.M. (2010). Functional delivery of viral miRNAs via exosomes. Proc. Natl. Acad. Sci. USA.

[22] Vechetti I.J., Peck B.D., Wen Y., Walton R.G., Valentino T.R., Alimov A.P., Dungan C.M., Van Pelt D.W., von Walden F., Alkner B. (2021). Mechanical overload-induced muscle-derived extracellular vesicles promote adipose tissue lipolysis. FASEB J..

[23] Elsherbini A., Bieberich E. (2018). Ceramide and Exosomes: A Novel Target in Cancer Biology and Therapy. Adv. Cancer Res..

[24] Chevillet J.R., Kang Q., Ruf I.K., Briggs H.A., Vojtech L.N., Hughes S.M., Cheng H.H., Arroyo J.D., Meredith E.K., Gallichotte E.N. (2014). Quantitative and stoichiometric analysis of the microRNA content of exosomes. Proc. Natl. Acad. Sci. USA.

[25] Helwa I., Cai J., Drewry M.D., Zimmerman A., Dinkins M.B., Khaled M.L., Seremwe M., Dismuke W.M., Bieberich E., Stamer W.D. (2017). A Comparative Study of Serum Exosome Isolation Using Differential Ultracentrifugation and Three Commercial Reagents. PLoS ONE.

[26] Mukherji S., Ebert M.S., Zheng G.X., Tsang J.S., Sharp P.A., van Oudenaarden A. (2011). MicroRNAs can generate thresholds in target gene expression. Nat. Genet..

[27] Llorente A., Skotland T., Sylvanne T., Kauhanen D., Rog T., Orlowski A., Vattulainen I., Ekroos K., Sandvig K. (2013). Molecular lipidomics of exosomes released by PC-3 prostate cancer cells. Biochim. Biophys. Acta.

[28] Pienimaeki-Roemer A., Kuhlmann K., Bottcher A., Konovalova T., Black A., Orso E., Liebisch G., Ahrens M., Eisenacher M., Meyer H.E. (2015). Lipidomic and proteomic characterization of platelet extracellular vesicle subfractions from senescent platelets. Transfusion.

[29] Wubbolts R., Leckie R.S., Veenhuizen P.T., Schwarzmann G., Mobius W., Hoernschemeyer J., Slot J.W., Geuze H.J., Stoorvogel W. (2003). Proteomic and biochemical analyses of human B cell-derived exosomes. Potential implications for their function and multivesicular body formation. J. Biol. Chem..

[30] De Gassart A., Geminard C., Fevrier B., Raposo G., Vidal M. (2003). Lipid raft-associated protein sorting in exosomes. Blood.

[31] Skotland T., Sagini K., Sandvig K., Llorente A. (2020). An emerging focus on lipids in extracellular vesicles. Adv. Drug Deliv. Rev..

[32] Trajkovic K., Hsu C., Chiantia S., Rajendran L., Wenzel D., Wieland F., Schwille P., Brugger B., Simons M. (2008). Ceramide triggers budding of exosome vesicles into multivesicular endosomes. Science.

[33] Skryabin G.O., Komelkov A.V., Savelyeva E.E., Tchevkina E.M. (2020). Lipid Rafts in Exosome Biogenesis. Biochemistry.

[34] Rabia M., Leuzy V., Soulage C., Durand A., Fourmaux B., Errazuriz-Cerda E., Koffel R., Draeger A., Colosetti P., Jalabert A. (2020). Bis(monoacylglycero)phosphate, a new lipid signature of endosome-derived extracellular vesicles. Biochimie.

[35] Skotland T., Sandvig K., Llorente A. (2017). Lipids in exosomes: Current knowledge and the way forward. Prog. Lipid Res..

[36] Skotland T., Ekroos K., Kauhanen D., Simolin H., Seierstad T., Berge V., Sandvig K., Llorente A. (2017). Molecular lipid species in urinary exosomes as potential prostate cancer biomarkers. Eur. J. Cancer.

[37] Baron C.L., Malhotra V. (2002). Role of diacylglycerol in PKD recruitment to the TGN and protein transport to the plasma membrane. Science.

[38] Alonso R., Rodriguez M.C., Pindado J., Merino E., Merida I., Izquierdo M. (2005). Diacylglycerol kinase alpha regulates the secretion of lethal exosomes bearing Fas ligand during activation-induced cell death of T lymphocytes. J. Biol. Chem..

[39] Cheng Q., Li X., Wang Y., Dong M., Zhan F.H., Liu J. (2018). The ceramide pathway is involved in the survival, apoptosis and exosome functions of human multiple myeloma cells in vitro. Acta Pharmacol. Sin..

[40] Sprong H., van der Sluijs P., van Meer G. (2001). How proteins move lipids and lipids move proteins. Nat. Rev. Mol. Cell Biol..

[41] Record M., Silvente-Poirot S., Poirot M., Wakelam M.J.O. (2018). Extracellular vesicles: Lipids as key components of their biogenesis and functions. J. Lipid Res..

[42] Record M., Carayon K., Poirot M., Silvente-Poirot S. (2014). Exosomes as new vesicular lipid transporters involved in cell-cell communication and various pathophysiologies. Biochim. Biophys. Acta.

[43] Barlow J.P., Solomon T.P. (2018). Do skeletal muscle-secreted factors influence the function of pancreatic beta-cells?. Am. J. Physiol. Endocrinol. Metab..

[44] Lee H.J., Lee J.O., Kim N., Kim J.K., Kim H.I., Lee Y.W., Kim S.J., Choi J.I., Oh Y., Kim J.H. (2015). Irisin, a Novel Myokine, Regulates Glucose Uptake in Skeletal Muscle Cells via AMPK. Mol. Endocrinol..

[45] Pedersen B.K., Fischer C.P. (2007). Beneficial health effects of exercise--the role of IL-6 as a myokine. Trends Pharmacol. Sci..

[46] Cui S., Sun B., Yin X., Guo X., Chao D., Zhang C., Zhang C.Y., Chen X., Ma J. (2017). Time-course responses of circulating microRNAs to three resistance training protocols in healthy young men. Sci. Rep..

[47] Fry C.S., Kirby T.J., Kosmac K., McCarthy J.J., Peterson C.A. (2017). Myogenic Progenitor Cells Control Extracellular Matrix Production by Fibroblasts during Skeletal Muscle Hypertrophy. Cell Stem Cell.

[48] Hudson M.B., Woodworth-Hobbs M.E., Zheng B., Rahnert J.A., Blount M.A., Gooch J.L., Searles C.D., Price S.R. (2014). miR-23a is decreased during muscle atrophy by a mechanism that includes calcineurin signaling and exosome-mediated export. Am. J. Physiol. Cell Physiol..

[49] Lovett J.A.C., Durcan P.J., Myburgh K.H. (2018). Investigation of Circulating Extracellular Vesicle MicroRNA Following Two Consecutive Bouts of Muscle-Damaging Exercise. Front. Physiol..

[50] Nielsen S., Scheele C., Yfanti C., Akerstrom T., Nielsen A.R., Pedersen B.K., Laye M.J. (2010). Muscle specific microRNAs are regulated by endurance exercise in human skeletal muscle. J. Physiol..

[51] Oliveira G.P., Porto W.F., Palu C.C., Pereira L.M., Petriz B., Almeida J.A., Viana J., Filho N.N.A., Franco O.L., Pereira R.W. (2018). Effects of Acute Aerobic Exercise on Rats Serum Extracellular Vesicles Diameter, Concentration and Small RNAs Content. Front. Physiol..

[52] Guigni B.A., van der Velden J., Kinsey C.M., Carson J.A., Toth M.J. (2020). Effects of conditioned media from murine lung cancer cells and human tumor cells on cultured myotubes. Am. J. Physiol. Endocrinol. Metab..

[53] Duong P., Chung A., Bouchareychas L., Raffai R.L. (2019). Cushioned-Density Gradient Ultracentrifugation (C-DGUC) improves the isolation efficiency of extracellular vesicles. PLoS ONE.

[54] Li K., Wong D.K., Hong K.Y., Raffai R.L. (2018). Cushioned-Density Gradient Ultracentrifugation (C-DGUC): A Refined and High Performance Method for the Isolation, Characterization, and Use of Exosomes. Methods Mol. Biol..

[55] Filipe V., Hawe A., Jiskoot W. (2010). Critical evaluation of Nanoparticle Tracking Analysis (NTA) by NanoSight for the measurement of nanoparticles and protein aggregates. Pharm. Res..

[56] Wu Y., Deng W., Klinke D.J. (2015). Exosomes: Improved methods to characterize their morphology, RNA content, and surface protein biomarkers. Analyst.

[57] Thery C., Amigorena S., Raposo G., Clayton A. (2006). Isolation and characterization of exosomes from cell culture supernatants and biological fluids. Curr. Protoc. Cell Biol..

[58] Zhong L., Kong J.N., Dinkins M.B., Leanhart S., Zhu Z., Spassieva S.D., Qin H., Lin H.P., Elsherbini A., Wang R. (2018). Increased liver tumor formation in neutral sphingomyelinase-2-deficient mice. J. Lipid Res..

[59] Mohamed A., Molendijk J., Hill M.M. (2020). lipidr: A Software Tool for Data Mining and Analysis of Lipidomics Datasets. J. Proteome Res..

[60] O’Donnell V.B., Dennis E.A., Wakelam M.J.O., Subramaniam S. (2019). LIPID MAPS: Serving the next generation of lipid researchers with tools, resources, data, and training. Sci. Signal..

[61] Dieterle F., Ross A., Schlotterbeck G., Senn H. (2006). Probabilistic quotient normalization as robust method to account for dilution of complex biological mixtures. Application in 1H NMR metabonomics. Anal. Chem..

[62] Smyth G.K. (2004). Linear models and empirical bayes methods for assessing differential expression in microarray experiments. Stat. Appl. Genet. Mol. Biol..

[63] Hagve T.A. (1988). Effects of unsaturated fatty acids on cell membrane functions. Scand. J. Clin. Lab. Investig..

[64] Ariyama H., Kono N., Matsuda S., Inoue T., Arai H. (2010). Decrease in membrane phospholipid unsaturation induces unfolded protein response. J. Biol. Chem..

[65] Zhang Y., Liu Y., Liu H., Tang W.H. (2019). Exosomes: Biogenesis, biologic function and clinical potential. Cell Biosci..

[66] Mathieu M., Névo N., Jouve M., Valenzuela J.I., Maurin M., Verweij F., Palmulli R., Lankar D., Dingli F., Loew D. (2020). Specificities of exosome versus small ectosome secretion revealed by live intracellular tracking and synchronized extracellular vesicle release of CD9 and CD63. bioRxiv.

[67] Nishida-Aoki N., Izumi Y., Takeda H., Takahashi M., Ochiya T., Bamba T. (2020). Lipidomic Analysis of Cells and Extracellular Vesicles from High- and Low-Metastatic Triple-Negative Breast Cancer. Metabolites.

[68] Dinkins M.B., Wang G., Bieberich E. (2017). Sphingolipid-Enriched Extracellular Vesicles and Alzheimer’s Disease: A Decade of Research. J. Alzheimers Dis..

[69] Pollet H., Conrard L., Cloos A.S., Tyteca D. (2018). Plasma Membrane Lipid Domains as Platforms for Vesicle Biogenesis and Shedding?. Biomolecules.

[70] Brzozowski J.S., Jankowski H., Bond D.R., McCague S.B., Munro B.R., Predebon M.J., Scarlett C.J., Skelding K.A., Weidenhofer J. (2018). Lipidomic profiling of extracellular vesicles derived from prostate and prostate cancer cell lines. Lipids Health Dis..

[71] Carrasco S., Merida I. (2007). Diacylglycerol, when simplicity becomes complex. Trends Biochem. Sci..

[72] Erion D.M., Shulman G.I. (2010). Diacylglycerol-mediated insulin resistance. Nat. Med..

[73] Bergman B.C., Hunerdosse D.M., Kerege A., Playdon M.C., Perreault L. (2012). Localisation and composition of skeletal muscle diacylglycerol predicts insulin resistance in humans. Diabetologia.

[74] Summers S.A., Chaurasia B., Holland W.L. (2019). Metabolic Messengers: Ceramides. Nat. Metab..

[75] Yuyama K., Sun H., Mikami D., Mioka T., Mukai K., Igarashi Y. (2020). Lysosomal-associated transmembrane protein 4B regulates ceramide-induced exosome release. FASEB J..

[76] Kosaka N., Iguchi H., Yoshioka Y., Takeshita F., Matsuki Y., Ochiya T. (2010). Secretory mechanisms and intercellular transfer of microRNAs in living cells. J. Biol. Chem..

[77] Gosejacob D., Jager P.S., Vom Dorp K., Frejno M., Carstensen A.C., Kohnke M., Degen J., Dormann P., Hoch M. (2016). Ceramide Synthase 5 Is Essential to Maintain C16:0-Ceramide Pools and Contributes to the Development of Diet-induced Obesity. J. Biol. Chem..

[78] Mebarek S., Komati H., Naro F., Zeiller C., Alvisi M., Lagarde M., Prigent A.F., Nemoz G. (2007). Inhibition of de novo ceramide synthesis upregulates phospholipase D and enhances myogenic differentiation. J. Cell Sci..

[79] Fekry B., Jeffries K.A., Esmaeilniakooshkghazi A., Szulc Z.M., Knagge K.J., Kirchner D.R., Horita D.A., Krupenko S.A., Krupenko N.I. (2018). C16-ceramide is a natural regulatory ligand of p53 in cellular stress response. Nat. Commun..

