# Supplementary material for: Skeletal Muscle Cell Growth Alters the Lipid Composition of Extracellular Vesicles
Source: Membranes (Basel). 2021 Aug 12;11(8):619. doi: 10.3390/membranes11080619 (PMC8397976; doi:10.3390/membranes11080619)
Supplement: Supplementary file 1 [file membranes-11-00619-s001.zip › membranes-1296930-supplementary.pdf]

membranes

MDPI

Supplementary Material

Skeletal Muscle Cell Growth Alters the Lipid Composition of Extracellular Vesicles

Taylor R. Valentino [1], Blake D. Rule [2], C. Brooks Mobley [1], Mariana Nikolova-Karakashian [1] and Ivan J. Vechetti [2,*]

[1] Department of Physiology, College of Medicine, Lexington, KY 40536, USA; trvalentino@uky.edu (T.R.V.); c.brooks.mobley@uky.edu (C.B.M.); mariana.karakashian@uky.edu (M.N.-K.)
[2] Department of Nutrition and Health Sciences, College of Education and Human Sciences, University of Nebraska-Lincoln, Lincoln, NE 68583, USA; brule2@unl.edu
* Correspondence: ivechetti@unl.edu; Tel.: +402-472-3716; Fax: +402-472-2895

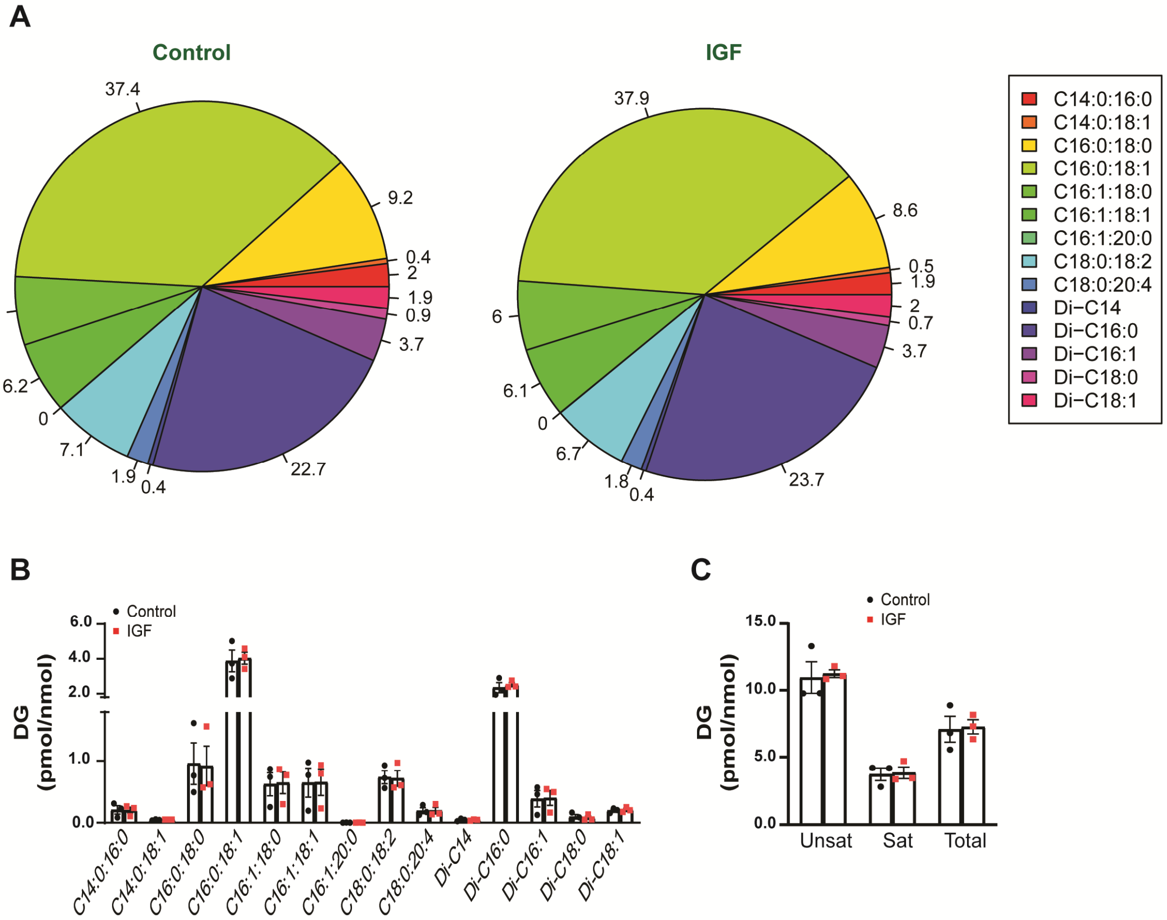

Figure S1. Diacylglycerol (DG) composition of C2C12 myotube cells. **A-** Pie chart highlighting DG species between myotube control (control) and IGF-treated myotubes (Treated); **B-** DG species between groups normalized for the amount of lipid-associated phosphate (a measure of total phospholipids in each preparation); **C-** Unsaturated, saturated and total DG between control and treated cells. Data is presented as percentage (A) and as mean ± SE (B-C) (n=3) with significance denoted by an asterisk compared to Control.

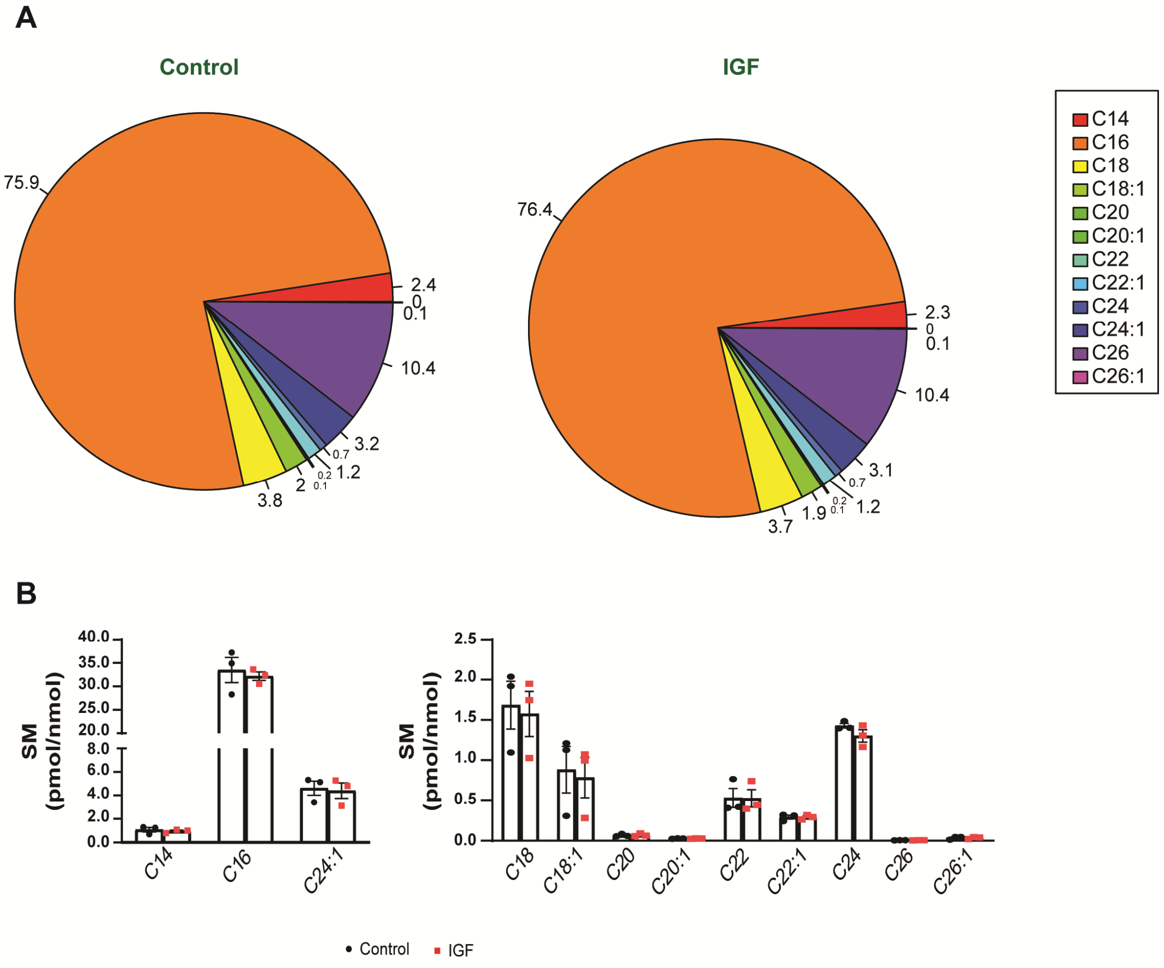

Figure S2. Sphingomyelin (SM) composition of C2C12 myotube cells. A- Pie chart highlighting SM species between myotube control (control) and IGF-treated myotubes (Treated); B- SM species between groups normalized for the amount of li-pid-associated phosphate (a measure of total phospholipids in each preparation). Data is presented as percentage (A) and as mean ± SE (B) (n=3) with significance denoted by an asterisk compared to Control.

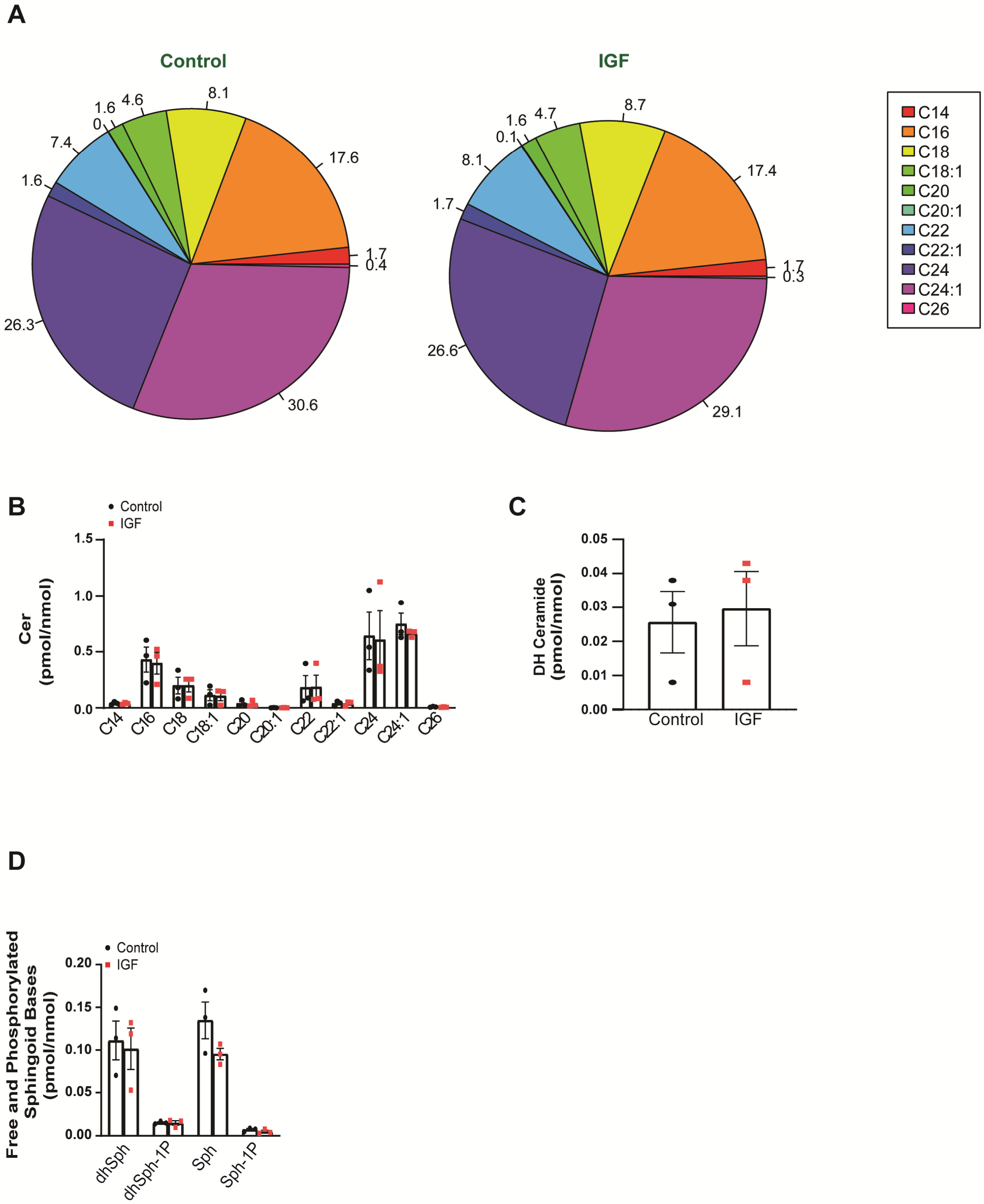

Figure S3. Ceramide (Cer) composition of C2C12 myotube cells. **A-** Pie chart highlighting Cer species between myotube control (control) and IGF-treated myotubes (Treated); **B-** Cer species between groups normalized for the amount of lipid-associated phosphate (a measure of total phospholipids in each preparation); Total amount of dihydro-ceramide (DH) (**C**) and sphingoid bases (**D**) between Control and Treated cells. dhSph: dihydrosphingosine, dhSph-1P: dihydrosphingosine-phosphate, Sph: sphingosine; Sph-1P: sphingosine-phosphate. Data is presented as percentage (A) and as mean ± SE (B-D) (n=3) with significance denoted by an asterisk compared to Control.
